# Molecular and cytogenetic dissection of stripe rust resistance gene *Yr83* from rye 6R and generation of resistant germplasm in wheat breeding

**DOI:** 10.3389/fpls.2022.1035784

**Published:** 2022-10-10

**Authors:** Guangrong Li, Jianbo Li, Yao Zhang, Qiang Ma, Ennian Yang, Peng Zhang, Ian Dundas, Zujun Yang

**Affiliations:** ^1^ Center for Informational Biology, School of Life Science and Technology, University of Electronic Science and Technology of China, Chengdu, China; ^2^ School of Life and Environmental Sciences, Plant Breeding Institute, The University of Sydney, Cobbitty, NSW, Australia; ^3^ Characteristic Crops Research Institute, Chongqing Academy of Agricultural Sciences, Yongchuan, China; ^4^ Crop Research Institute, Sichuan Academy of Agricultural Sciences, Chengdu, China; ^5^ School of Agriculture, Food and Wine, The University of Adelaide, Waite, Glen Osmond, SA, Australia

**Keywords:** FISH, rye, wheat, stripe rust, *Yr83*

## Abstract

Rye 6R-derived stripe rust resistance gene *Yr83* in wheat background was physically mapped to fraction length (FL) 0.87-1.00 on the long arm by non-denaturing-fluorescence *in situ* hybridization (ND-FISH), Oligo-FISH painting and 6R-specific PCR markers.Stripe rust resistance gene *Yr83* derived from chromosome 6R of rye (*Secale cereale*) “Merced” has displayed high resistance to both Australian and Chinese wheat stripe rust isolates. With the aim to physically map *Yr83* to a more precise region, new wheat- 6R deletion and translocation lines were produced from derived progenies of the 6R(6D) substitution line. The non-denaturing fluorescence *in situ* hybridization (ND-FISH) patterns of 6R were established to precisely characterize the variations of 6R in different wheat backgrounds. Comparative ND-FISH analysis localized the breakpoints of 6RL chromosomes relative to Oligo-pSc200 and Oligo-pSc119.2 rich sites in deletion lines. Molecular marker and resistance analyses confirmed that *Yr83* is physically located at the fraction length (FL) 0.87-1.00 of 6RL and covers the corresponding region of 806-881 Mb in the reference genome of Lo7. Oligo-FISH painting demonstrated that the region carrying *Yr83* is syntenic to the distal end of long arm of homoeologous group 7 of the Triticeae genome. The developed wheat-6R lines carrying the *Yr83* gene will be useful for breeding for rust resistance.

## Introduction

Stripe rust (yellow rust), caused by *Puccinia striiformis* f. sp. *tritici* (*Pst*), is an important disease that occurs in most global wheat-growing regions (Morgounov et al., 2012; Wang and Chen, 2017). Cultivation of resistant cultivars is economical and environmentally friendly compared to chemical control. Eighty-four stripe rust resistance genes have been identified and many are being used in wheat breeding (McIntosh et al., 2021; Klymiuk et al., 2022). However, rapid emergence of virulent Pst races has rendered many resistance genes in released cultivars ineffective, which has prompted the continuing search for more durable resistance (Kou and Wang, 2010; Schwessinger, 2017). Breeding durable disease resistant cultivars relies largely on continually discovering and introducing new resistance genes into adapted varieties, especially the genes from wild species (Ellis et al., 2014; Zhang et al., 2018).

Cultivated rye (*Secale cereale* L.) has been a valuable source of potentially useful genes for wheat improvement (Rabinovich, 1997; Tang et al., 2011). However, reports on stripe rust resistance genes derived from *Secale* species are far behind the other foliar disease resistance genes identified (Spetsov and Daskalova, 2022). The chromosome arm 1RS from ‘Petkus’ rye was introduced into many wheat cultivars grown worldwide and carries stripe rust resistance gene *Yr9* (Mago et al., 2005), which is no longer effective in most locations. Several rye chromosomes were found to have novel stripe rust resistance and have been incorporated into wheat backgrounds (Wang et al., 2009; Li et al., 2016; Schneider et al., 2016; An et al., 2019). Among them, chromosome 6R has displayed a number of polymorphic variations, and the 6R derived from different origins has been found to possess multiple disease-resistance genes. Several wheat-6R introgression lines with novel disease resistance gene(s) have been developed (Friebe et al., 1996; Dundas et al., 2001; An et al., 2015; Hao et al., 2018; Li et al., 2020b; Duan et al., 2022; Ashraf et al., 2022).

Asiedu et al. (1990) developed a wheat-chromosome 6R substitution line Sub6R(6D) from triticale T-701, derived from rye “Merced”, and later Dundas et al. (2001) localized the nematode resistance gene *CreR* to an interstitial region in the long arm of 6R. Li et al. (2020b) found that the Sub6R(6D) were highly resistant to both stripe rust and powdery mildew pathogens, and physically located the new stripe rust resistance gene *Yr83* on FL 0.73-1.00 of 6RL by FISH on wheat-rye 6R translocation and deletion lines. In this study, the physical location of *Yr83* on 6RL was further narrowed down by molecular cytogenetic approaches with the information from the available whole genomic sequences of *Secale* genomes (Bauer et al., 2017; Li et al., 2021; Rabanus-Wallace et al., 2021).

The objectives of this study were to identify new wheat - 6R deletion and translocation lines and further physically localize *Yr83* gene on the specific region by FISH and molecular markers, and assess the developed wheat-6R introgression lines for wheat breeding.

## Materials and methods

### Plant materials

The triticale line T-701 was originally developed by CIMMYT (Li et al., 2020b), while the wheat-*S. cereale* 6R(6D) substitution line (Sub6R(6D)) and deletion line T6RL22 were developed at University of Adelaide, Australia (Asiedu et al., 1990; Dundas et al., 2001; Li et al., 2020a). CS-Imperial rye 6R addition line was obtained from Dr. Bernd Friebe, Wheat Genetics Resource Center, Department of Plant Pathology, Kansas State University, USA. *S. cereale* cv. Kustro and Weining were kindly provided by Prof. Zongxiang Tang and Huaiyu Zhang at Sichuan Agricultural University, China; Wheat cv. Mianyang 11 (MY11) and Chuanmai 42 (CM42) are maintained in our laboratory at the School of Life Science and Technology, University of Electronic Science and Technology of China.

### Fluorescence *in situ* hybridization (FISH)

Root tips from germinated seeds were collected and treated with nitrous oxide followed by enzyme digestion, using the procedure of Han et al. (2006). Synthesized oligo-nucleotide probes Oligo-pSc200, Oligo-pSc119.2, and Oligo-pTa535 were used for identifying wheat chromosomes following the descriptions of Tang et al. (2014) and Fu et al. (2015). A new probe Oligo-248 was developed from tandem repeats database on B2DSC website (Lang et al., 2019). The tandem repeat-based oligo-nucleotide probes Oligo-k288 (Wang et al., 2019) and Oligo-D (Tang et al., 2018) for ND-FISH are listed in **Table 1**. After FISH using the above oligos as probes, the sequential FISH painting with bulked oligos was conducted following the description by Li and Yang (2022). Photomicrographs of FISH chromosomes were taken with an Olympus BX-53 microscope equipped with a DP-70 CCD camera. Images were processed using Photoshop 3.0 (Adobe Systems Incorporated, CA, USA).

**Table 1:** The Oligo probes for chromosome identification in ND-FISH.

Name	Sequences	Chromosomes	References
Oligo-k288	CTTCATAGTCCGGGAGTCCGGCCAAAGGTCATAGTCCGGCCATCC	Wheat A B genomes	Wang et al., 2019
Oligo-D	TACGGGTGCCAAACGAGTGTCTGAAAGACTCCTCGAGAGGAAAATGCGAA	Wheat D genomes	Tang et al., 2018
Oligo-Ku	GATCGAGACTTCTAGCAATAGGCAAAAATAGTAATGGTATCCGGGTTCG	Rye	Fu et al., 2015
Oligo-pSc119.2	CCGTTTTGTGGACTATTACTCACCGCTTTGGGGTCCCATAGCTAT	Wheat, rye	Tang et al., 2014
Oligo-pTa535	AAAAACTTGACGCACGTCACGTACAAATTGGACAAACTCTTTCGGAGTATCAGGGTTTC	Wheat	Tang et al., 2014
Oligo-248	TAAACCCTACCACACGTCACTCTGAAACAAAGGTCGCCCAAGAACTCCT	Wheat, 6R	This study

### Molecular marker analysis

DNA was extracted from young leaves using a sodium dodecyl sulfate (SDS) protocol (Li et al., 2015). The PCR markers in rye (Qiu et al., 2016), 6R-specific markers (Li et al., 2016; Du et al., 2018), PCR-based Landmark Unique Gene (PLUG) primers (Ishikawa et al., 2009; Li et al., 2013), CINAU markers (Zhang et al., 2017), and rye Lo7 genomic region-specific markers were based on searching the website of the Triticeae Multi-omics Center (http://202.194.139.32/). All primers were synthesized by Shanghai Invitrogen Biotechnology Co. Ltd. Amplified PCR products were electrophoresed on a 1.0% agarose gel as described by Li et al. (2015). The physical locations of the molecular markers on chromosomes were based on the reference genomes of Chinese Spring wheat (IWGSC 2018) and Lo7 rye (Rabanus-Wallace et al., 2021).

### Stripe rust reactions

Stripe rust reactions were observed in the field at the Sichuan Academy of Agricultural Sciences Experimental Station. Ten plants were grown per 1-m row with a 25-cm spacing between rows. Bread wheat cv. MY11 planted on both sides of each experimental row served as an inoculum spreader and susceptible control after inoculation with a mixture of races CYR32, 33 and 34. Reactions evaluated at the heading and grain-filling stages were recorded on a 0–4 infection type (IT) scale according to Bariana and McIntosh (1993).

## Results

### Karyotyping of 6R in Sub6R(6D) and derived lines in Sichuan wheat background

**Figure 1:**
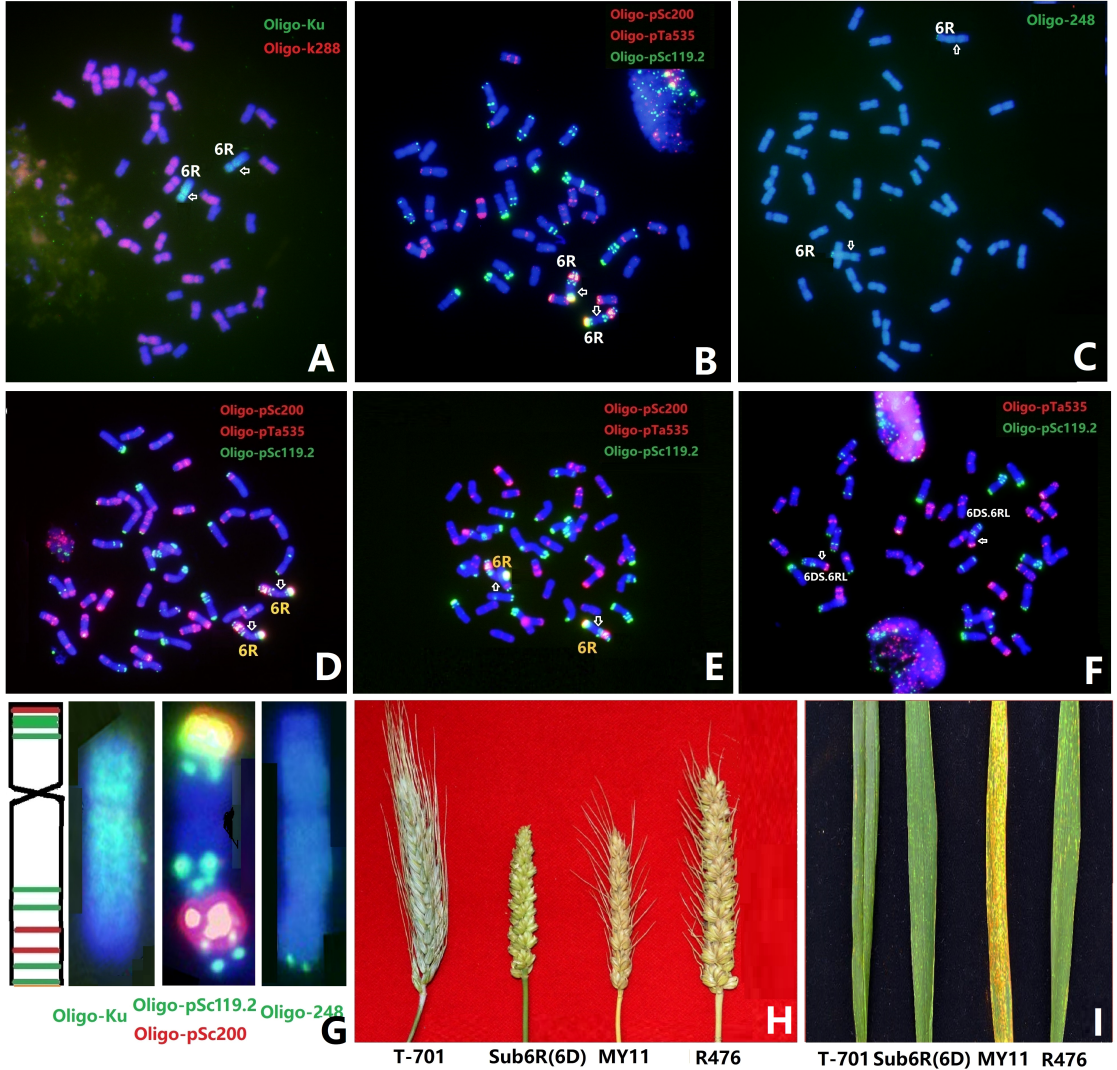
ND-FISH karyotyping of chromosome 6R in different wheat backgrounds. FISH of Sub6R(6D) line was performed using rye-specific oligo probe Oligo-Ku **(A)**, Oligo-pSc119.2+ Oligo-pSc535 + Olio-pSc200 **(B)**, and Oligo-238 **(C)**. Lines R323 in CM42 **(D)**, R476 in MY11 **(E)**, and R27 with T6DS.6RL in MY11 **(F)** were revealed by ND-FISH using probes Oligo-pSc119.2, Oligo-pTa535, and Olio-pSc200. The karyotype of 6R is shown using multiple probes **(G)**, the spike morphology **(H)** and stripe rust reaction **(I)** of the above wheat-6R lines and the parents are presented. Arrows pointed the positions of centromeres of 6R.

Li et al. (2020a) characterized chromosome 6R from Triticale T-701 and Sub6R(6D) by sequential multicolor FISH (mc-FISH) and genomic *in situ* hybridization (GISH). In the present study, ND-FISH using probe Oligo-Ku was used in place of GISH to identify the rye chromosome 6R in Sub6R(6D) (**Figure 1A** **)**. ND-FISH with probes Oligo-pSc119.2 and Oligo-pSc200 was used to generate the standard karyotype of 6R. The short arm of 6R carried a strong and a weak pSc119.2 sites at telomeric and subtelomeric regions, respectively, and a strong Oligo-pSc200 site in the distal region. The 6RL arm had four Oligo-pSc119.2 and two Oligo-pSc200 signal sites, and of these the Oligo-pSc200 hybridization sites were located between the interstitial and sub-telomeric Oligo-pSc119.2 sites (**Figure 1B**). Li et al. (2020a) observed two strong hybridization bands on 6RL shown by GISH using rye genomic DNA as probe. Based on the comparison of the GISH banding and FISH patterns (Zhou et al., 2010), the two GISH bands on 6RL were identical to the physical positions of Oligo-pSc200 hybridization sites. A line R23 containing 6DS.6RL translocation was selected from the BC_1_F_4_ generation from Sub6R(6D) to MY11. Moreover, we developed a new ND-FISH probe Oligo-248, which gives rise to a specific hybridization site at the telomeric region of 6RL in T-701, Sub6R(6D) and line R23 (**Figure 1**; **Figure S1**). The updated ND-FISH karyotype of 6R (**Figure 1G**) will be helpful to characterize structural variations for chromosome 6R, especially the distal end of 6RL, in the progenies or mutant lines.

**Figure 2:**
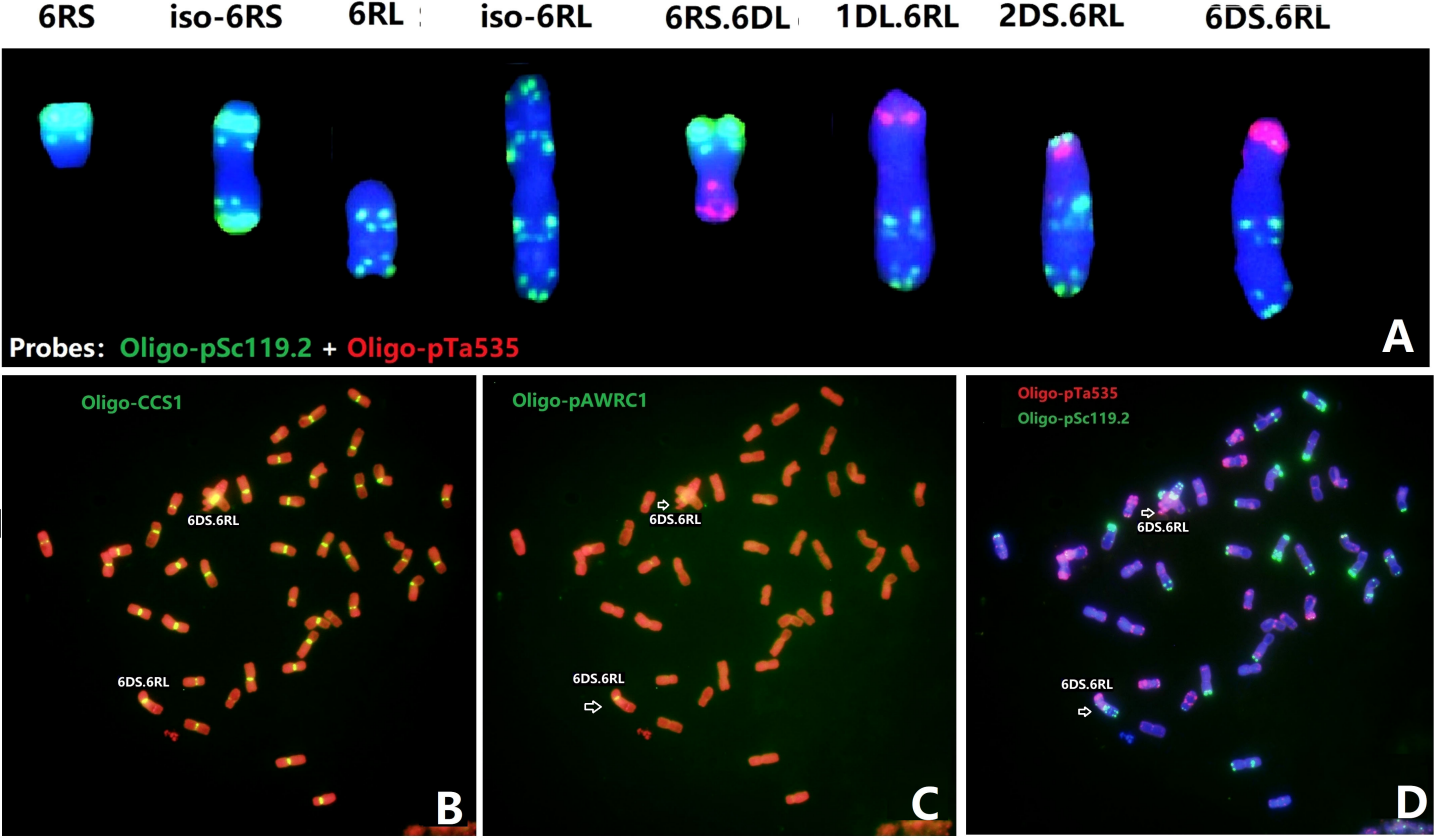
Sequential ND-FISH of the chromosome variations of 6R **(A)** and line R23 with T6DS.6RL in a MY11 background **(B-D)**. The different types of 6R variations and wheat-6R translocations are shown by ND-FISH with probes Oligo-pSc119.2 and Oligo-pTa535 **(A)**. The centromeric probes Oligo-CCS1 **(B)** and Oligo-pAWRC1.1 **(C)**, and Oligo-pSc119.2 + Oligo-pTa535 **(D)**, were used to show that the centromere in T6DS.6RL contains both wheat and rye centromeres.

With the aim to transfer segments of rye 6R into Sichuan wheat background, a total of 908 plants of F_3_ progenies between Sub6R(6D) and Sichuan wheat CM42 and MY11 were screened by ND-FISH using probes Oligo-pSc119.2 and Oligo-pSc200. Lines R323 and R476 with a pair of chromosomes 6R in CM42 and MY11 backgrounds were produced in the BC_1_F_3_ generation from Sub6R(6D) to CM42 and MY11, respectively. The ND-FISH karyotypes of the chromosomes of R323 and R476 are shown in **Figures 1D, F**, respectively. The telosomic chromosomes 6RS, 6RL, isochromosomes iso6RS and iso6RL were identified in the F_4_ progenies (**Figure 2A**). In addition, 25 plants (2.7%) had different wheat-6R translocations including T1DS.6RL, T2DS.6RL, T6DS.6RL, T6BS.6RL, and T6RS.6DL, indicating high frequency of breakage and re-fusion of 6R with wheat chromosomes. A homozygous T6DS.6RL translocation line R23 was developed (**Figure 2**). ND-FISH with the grass common centromeric repeat Oligo-CCS1 and rye-specific centromeric repeat Oligo-pAWRC1.1 probes (**Figures 2B–D**) suggested that line R23 contained a recombined centromere from wheat 6D and rye 6R based on different intensities of signals using the two probes. The line R476 displayed an earlier flowering time of 7-10 days and reduced plant height of 80-90 cm, with significant longer spike length compared to the parent MY11 (**Figure 1H**), in addition to retaining the stripe rust resistance from 6RL (**Figure 1I**), suggesting that “Merced” derived 6R has positive effects on agronomic traits and rust resistance in wheat background.

### Identification of new 6R deletion and translocation lines

**Figure 3:**
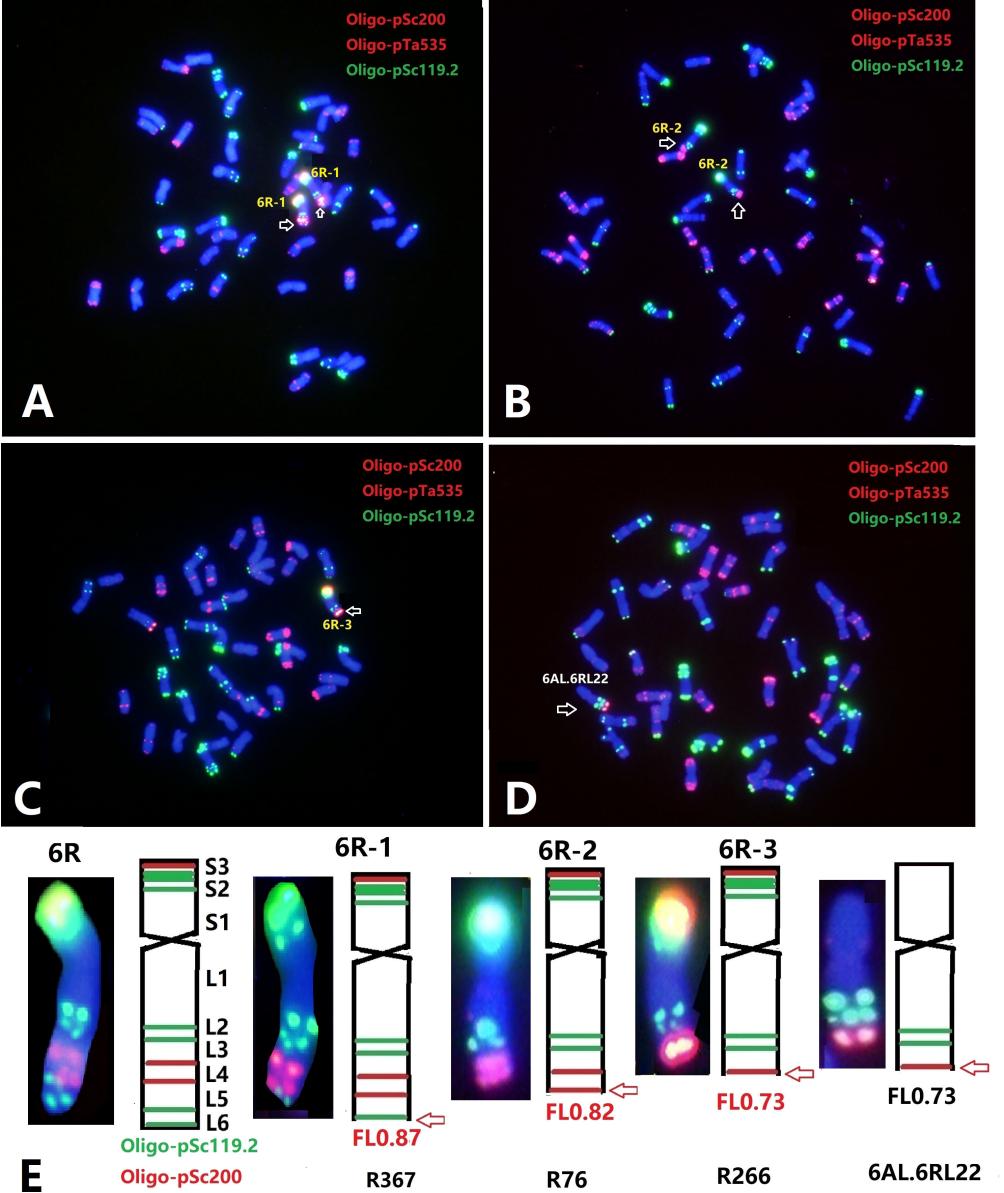
ND-FISH of wheat-rye 6R lines from irradiated progenies of Sub6R(6D) lines. The wheat-6R lines contains different types of 6RL deletions **(A–D)**. The diagrams show the FISH patterns of the 6RL deletions by ND-FISH of Oligo-pSc119.2 (green) + Oligo-pTa535 (red) + Oligo-pSc200 (red) as probes **(E)**.

With the aim to further localize the *Yr83* gene, a total of 1,662 M_3_ plants from 195 M_2:3_ families of the gamma-irradiated Sub6R(6D) line were screened by ND-FISH using Oligo-pSc119.2, Oligo-pTa535, and Oligo-pSc200. Approximately, 4.0% of plants contained a modified 6R, including deletion, telosomic or isotelosomic 6R, and wheat-6R translocation chromosomes. Based on the standard karyotype of 6R (**Figure 1**), three types of deletions in 6RL were detected based on FISH with Oligo-pSc119.2 and Oligo-pSc200. Type 6R-1 had a deletion showing the loss of the most distal Oligo-pSc119.2 site at the terminal end of 6RL with an estimated breakpoint at FL 0.87 on 6RL (**Figures 3A, E** **)**. Type 6R-2 was a larger deletion of the distal end of 6RL with the breakpoint after the second Oligo-pSc200 site (**Figures 3B, E** **)**, which was estimated at the breakpoint of FL 0.82, and the deleted segment was FL 0.82-1.00. Type 6R-3 was an even bigger deletion than Type 6R-2 with the breakpoint between the two Oligo-pSc200 sites (**Figures 3C, E** **)**. A line T6AL.6RLdel developed from progenies of the cross Sub6R(6D)/CS *ph1b* mutant Schomburgk/6R deletion (Dundas et al., 2001) was also characterized by ND-FISH with Oligo-pSc200, Oligo-pTa535, and Oligo-pSc119.2 (**Figure 3D**). The breakpoint was located between the two Oligo-pSc200 sites on 6RL, which is similar to that in Type 6R-3 (**Figure 3C**) with FL 0.73 (**Figure 3E**).

Sequential ND-FISH using probes Oligo-k288 or Oligo-D and Oligo-Ku (**Table 1**), as well as Oligo-pSc119.2 and Oligo-pTa535, was used to detect the breakpoints between wheat and 6R. Probe Oligo-k288 hybridizes specifically to the A and B-genome chromosomes, while Oligo-D hybridizes specifically to D-genome chromosomes. Two reciprocal Robertsonian translocations involving 7A-6R and 7D-6R were observed in 0.06% plants, while 12 types of non-Robertsonian wheat-6R translocations were detected in 2.13% plants (**Figure S2**). The breakpoints at FL 0.50 of 6RS, and FL 0.50 and FL 0.73 on 6RL occurred at the highest frequencies for the non-Robertsonian translocations with wheat chromosomes.

### Confirmation of the breakpoints on 6RL deletions by PCR markers

**Figure 4:**
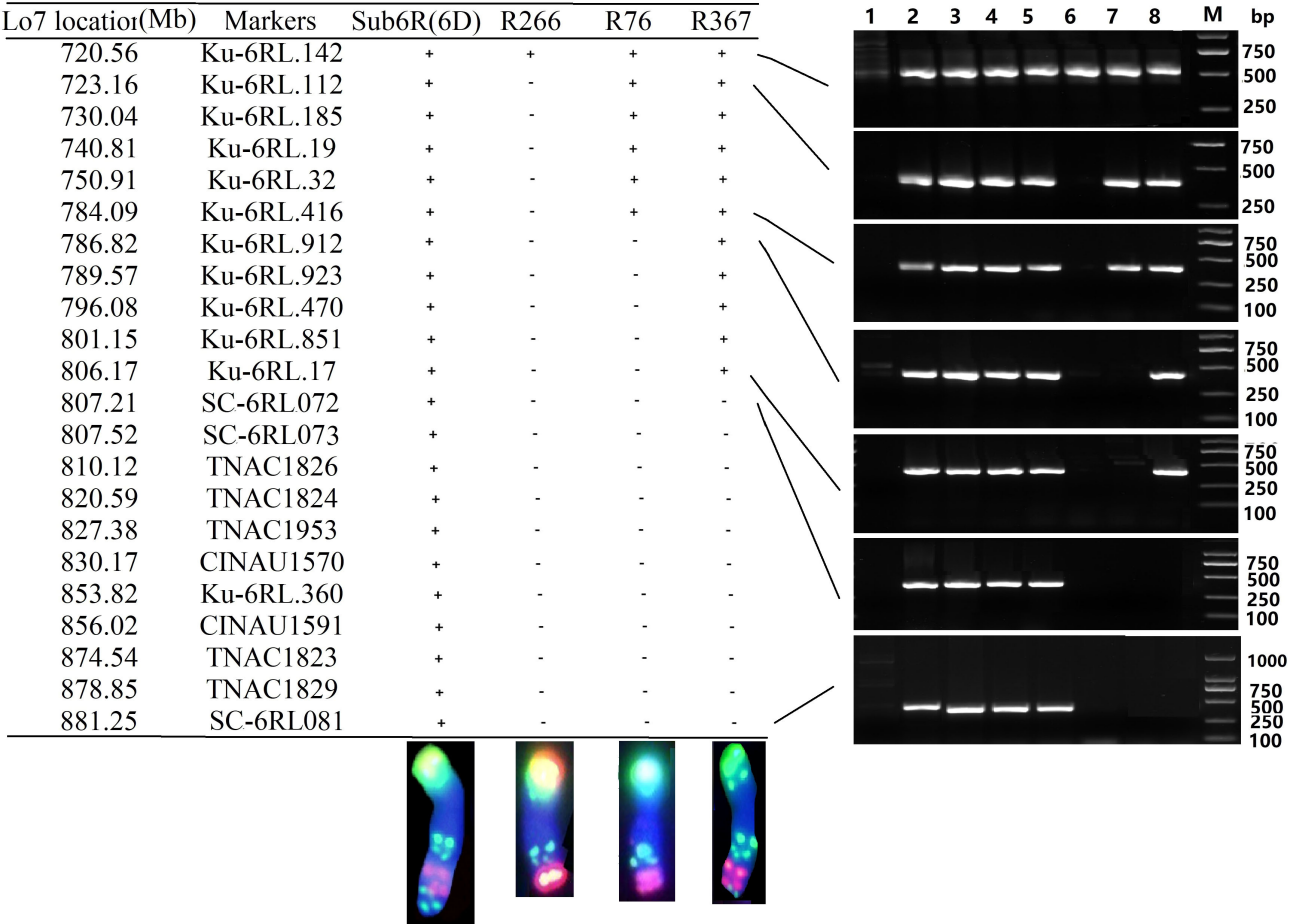
Physical locations of chromosome-specific markers on Sub6R(6D), 6RL deletion lines R266, R76, and R376. The marker location (left) was determined in reference to the genome of Lo7 (Mb). “+” represents amplification, while “-” represents no amplification of 6RL specific bands. The plant materials 1-8 used (right) were CS, Weining rye, T-701, Sub6R(6D), R23, R266, R76, and R367. The chromosomes were subjected to ND-FISH using Oligo-pS119.2 (green) and Oligo-pSc200 (red).

In total, 190 PLUG markers (Ishikawa et al., 2009), 321 CINAU markers (Zhang et al., 2017), and 190 markers specific for 6RL in Kustro rye (Qiu et al., 2016) were used to amplify DNA from the wheat lines MY11, Sub6R(6D), T6DS.6RL, R266, R76 and R367. The additional 16 6RL specific primers (**Table 2**) were designed with reference to the location of genome sequence of Lo7 6RL (Rabanus-Wallace et al., 2021). As shown in **Figure 4**, the breakpoints in R266 (and T6AL.6RL22) were physically located in the region between 720.56-723.16 Mb by markers Ku-6RL142 and Ku-6RL112. The breakpoint of R76 was physically located in the region between 784.09-786.82 Mb by markers Ku-6RL416 and Ku-6RL912. The breakpoint of R367 was physically located 806.17-807.21 Mb by markers Ku-6RL17 and SC-6RL072 (**Figure 4**). In the distal region of 6RL, markers SC-6RL082 to SC-6RL087 (**Table 2**) did not amplify in Sub6R(6D), but amplified in CS-Imperial 6R addition. It is likely that the present 6R may have lost the region of 882-885 Mb at terminal end of 6RL compared to Lo7 rye genome or possibly the two rye cultivars were highly divergent in the region. The association between the unique hybridization of probe Oligo-248 in ND-FISH (**Figure S1**) and the apparent loss of the 882-885 Mb region of 6RL needs to be further studied. The deletion in R367 was thus about 75 Mb of 6RL (806–881 Mb), corresponding to the reference genome of Lo7.

**Table 2:** The 6RL-specific markers used on Sub6R(6D).

Primers	Forward	Reverse	Location on Lo7 (Mb)	Amplification of 6R
SC-6RL072	ATGGAGTTGAAGAAGTTCCG	CTGTCAGAAAGCAGTAATAATAC	807.20	**+**
SC-6RL073	CACAAAGGATATAGGTGCCT	CTGTGCTATTTGTGGGCTGC	807.50	**+**
SC-6RL074	TCGGTGCCCAATTTCTCTG	GTAGATGATAGGCTCCTCCA	808.20	**+**
SC-6RL075	GCTACGATGGCTAAGAAGGT	TGCAATTCCAACCACTGATC	810.00	**+**
SC-6RL076	GCACAAGATGTAGCCAGCTG	GCACGTAATTTGGCGATGG	874.74	**+**
SC-6RL077	CATCTCTTCAGCGTTATAAGG	AGTACTGCATCTATAATGGTG	875.99	**+**
SC-6RL078	GTGTTCAGACAGAGGTTAG	CTACTAAGTTGATGTCAGCT	877.93	**+**
SC-6RL079	AACGTCAACAATTGGTTCGG	GAGCCAAGGCTATACACTAATC	879.49	**+**
SC-6RL080	CGGAACTAAATGCTCATGTC	GCCGTGTCTGACAGTCTTTC	880.07	**+**
SC-6RL081	CTTCGTCGACTTGGACATATTG	GGTTGGCAATTGGCATATATC	881.25	**+**
SC-6RL082	ATGACCTGATCCCTGCAACAC	ATCGATGCAGGATCAGCAGC	882.74	**-**
SC-6RL083	GCGACACCATTAACCTGTGG	CCAGGACTGAGTACTATTACAG	883.80	**-**
SC-6RL084	CCATCGTAAGTACTGCATCGT	GTGTAATCTAGAGAAAGGTGGC	884.41	**-**
SC-6RL085	GTCACCGTGGTCTATATCGACA	CCTCGGGTCCAACATTTAGAG	884.61	**-**
SC-6RL086	CTTAGTTAGACAGAGTGCGGTG	GAGGTCCACTAAGTAAGTTTC	884.77	**-**
SC-6RL087	AGGGGTATGTAAGCTATGCT	CTGCCCTGATCCTAATGACTAC	884.82	**-**
P60*	TGGGGAATAGCTGGCATTGG	TCGGTAGGGTAGACGGTGAG	865.17	**+**
P70*	AATGGGAGGACTCTTGCGTG	CTGGGAATGAACCGACAGCT	880.05	**+**

*Primers P60 and P70 are referred Duan et al. (2022).

**Figure 5:**
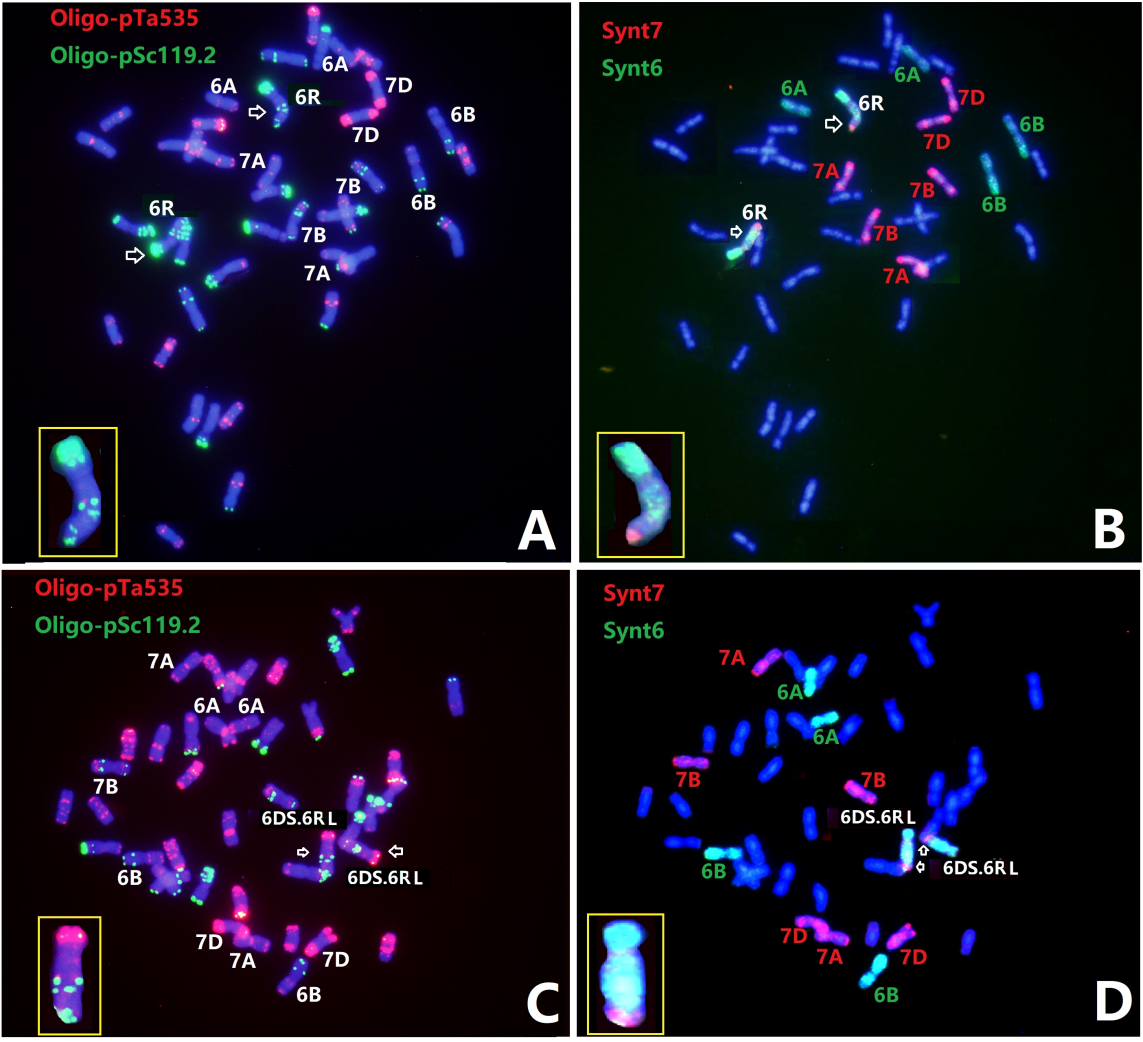
ND-FISH and Oligo-FISH painting of lines Sub6R(6D) **(A, B)** and R23 **(C, D)**. Probes Oligo-pSc119.2 + Oligo-pTa535 **(A, C)** and Synt6 + Synt7 **(B, D)** were used. The Synt7 hybridization sites corresponded to the distal two Oligo-pSc119.2 sites in 6RL.

Line Sub6R(6D) and T6DS.6RL translocation line R23 were analyzed with ND-FISH using Oligo-pSc119.2 and Oligo-pTa535 as probes, and then FISH painting with the bulk oligo probes Synt6 and Synt7 **(** **Figure 5** **)**. FISH painting with Synt6 showed that the entire 6RS and a proximal region of 6RL had distinct signals, suggesting homoeology to the wheat group 6 chromosomes. Comparing the karyotype of 6RL by ND-FISH with probes Oligo-pSc119.2 and Oligo-pTa535, it seems that the tandem repetitive sequence localizations of the two Oligo-pSc119.2 sites were likely to be in the Synt7 regions in 6RL of Sub6R(6D) and R23. The FISH painting with probe Synt7 hybridized to the distal region of 6RL at about FL 0.82-1.00, indicating that this region is homoeologous to wheat group 7 chromosomes.

### Stripe rust resistance and physical location of *Yr83*

When inoculated with a mixture of *Pst* races (CYR32, CYR33, and CYR34) in the field, the wheat parents CM42 and MY11 were susceptible, while line Sub6R(6D) and T6DS.6RL line R23 were resistant. The homozygous 6RL deletion lines R266, R76 and R367 were susceptible to *Pst* at adult plant stage (**Figure S3**). The results indicated that the stripe rust resistance *Yr83* in 6RL was located in FL 0.87-1.00, which corresponded to 806.26-881.00 Mb region in the rye genome of Lo7.

## Discussion

Over 21,000 rye accessions are maintained in genebanks worldwide, and of these approximately 35% are landraces and wild species (FAO, 2010). Hexaploid triticale contains wheat and rye genomes, and shows relatively superior vigor and reproductive stability (Lukaszewski and Gustafson, 1987; Cheng and Murata, 2002). Studies on genome-wide diversity have revealed diversified genetic structures in rye and Triticale germplasm (Bolibok-Bragoszewska et al., 2014; Sun et al., 2022; Cao et al., 2022). Among the rye chromosomes, the *S. cereale* chromosome 6R has displayed rapid structural change and high levels of heterogeneity of heterochromatin blocks, which has been revealed by FISH using tandem repeat probes on different rye cultivars (Guo et al., 2019). The FISH and GISH patterns using probes pSc119.2 and pSc200 showed three different karyotypes of 6R (**Figure S4**). The FISH patterns of 6R in Weining rye (Li et al., 2021) and Kustro rye (Li et al., 2016) had four pSc119.2 and two pSc200 sites, which is identical to the 6R in Sub6R(6D) from Merced rye (Type 6R-I, **Figure S4**). Two hybridization sites of pSc119.2 and three sites of pSc200 sites were observed on the 6RL of YT2 (Han et al., 2022), which is similar to the FISH patterns of CS-Imperial 6R (Type 6R-II, **Figure S4**). Three pSc119.2 and two pSc200 sites on 6RL were observed in Jinzhouheimai (Wu et al., 2018), AR106BONE (Duan et al., 2022), and rye cv. Qinling (Hao et al., 2018), which is typical of 6R-III (**Figure S4**). In addition, our previous study on *S. africanum* 6R^afr^ displayed a completely different karyotype compared to cultivated rye 6R (Li et al., 2020a), which has three pSc119.2 sites and a lack of pSc200 hybridization (**Figure S4**). Probe Oligo-248 was found to specifically hybridize to the 6RL terminal region of T-701, Sub6R(6D) and the T6DS.6RL line R23 (**Figure 1** and **Figure S1**). The same pattern was also observed on 6RL in rye AR106BONE, but not in Imperial, Kustro, or Weining rye. Therefore, both molecular and cytogenetic evidence suggests that different sources of 6RL may provide diversity for future transfer of novel genetic variation to wheat.

The 6R chromosomes from different cultivated rye genotypes contain disease resistance genes with potential for wheat improvement. The 6RL chromosomes from rye cv. Prolific (Heun and Friebe, 1990; Friebe et al., 1994), JZHM (Wang et al., 2010), German White (An et al., 2015; Han et al., 2022), and Kustro (Li et al., 2016; Du et al., 2018) have powdery mildew resistance genes. Wu et al. (2018) localized PmJZHM6RL with 12 molecular markers to FL 0.51-1.00 of 6RL. Hao et al. (2018) reported the powdery mildew gene *Pm56* on 6RS from rye cv. Qinling. Han et al. (2022) located the *PmYT2* gene in the sub-telomeric region of 6RL, the physical region being at the 67 Mb interval corresponding to 890.09–967.51 Mb in Weining rye and 784-836 Mb in Lo7. The breakpoint at 784 Mb of Lo7 is similar to that in R76 in this study, about FL 0.82 of 6RL (**Figure 4**). The allelism between the *Pm* gene in “Merced” rye and *Pm20* (Friebe et al., 1994), *PmYT2* (Han et al., 2022) and *PmJZHM* (Wang et al., 2010) need to be tested. With respect to the stripe rust resistance, the Imperial rye 6R added in Chinese Spring and the Kustro rye 6R derived addition line 18T142 were both susceptible to stripe rust races CYR32-34 (ZX Tang, personnel communication). Li et al. (2020a) localized *Yr83* on 6RL FL 0.73-1.00 using Sub6R(6D) derived deletion lines that conferred stripe rust resistance to multiple Chinese and Australian pathotypes. The present study further localized the *Yr83* gene on the FL 0.87-1.00 of 6RL, which corresponds to 806-881 Mb in 6R of Lo7. Recently, Duan et al. (2022) physically located a fragment of about 37 Mb (corresponding to 848-885 Mb of Lo7) in the telomeric region of 6RL derived from rye line AR106BONE carrying *Yr6R^Ar^L* with high resistance to stripe rust CYR34. The allelism between *Yr83* and *Yr6R^Ar^L* needs to be tested. Ashraf et al. (2022) identified a new stripe rust gene *YrSLU* on a small translocation of T6DS.6DL.6RL.6DL from 6RL of rye SLU126, and mapped it in the terminal region of 6RL. Our previous identified *S. africanum* 6R^afr^ derived stripe rust resistance gene was located on FL 0.95-1.00 of 6R^afr^S (Li et al., 2020a). These 6R chromosomes from cultivated and wild rye carrying stripe rust resistances are worthwhile for further wheat-rye introgression and germplasm enhancement.

Chromosome region-specific markers and the recently complete genomic sequences of rye are valuable resources for targeting introgressed chromatin from different cultivated and wild rye accessions to wheat (Tang et al., 2011; Qiu et al., 2016; Li et al., 2016; Li et al., 2021; Rabanus-Wallace et al., 2021). The evidence supports that the present-day cultivated rye 6RL contains the translocated segments from 3R and 7R, and these chromosome re-arrangements were clearly defined by comparative molecular markers (Devos et al., 1993; Li et al., 2013; Rabanus-Wallace et al., 2021). The centromeric regions of 6R were predicted at 290-300 Mb region of Lo7 genome by the physical location of pAWRC1.1 repeats using B2DSC web site (Lang et al., 2019). Li et al. (2020a) located *Yr83* on FL 0.73-1.00 of 6RL, which is the distal 27% of the 6RL arm. The present study demonstrated this breakpoint to be at 720.56-723.16 Mb. The markers revealed that the FL 0.73-1.00 of 6RL included the homoeologous groups 3 and 7 re-arranged regions (Li et al., 2020a). Our study produced a deletion line R376 with a breakpoint at about 805 Mb of Lo7 genome, which was at FL 0.87-1.00 of 6RL, and Oligo-FISH painting confirmed that the fragment of the last two Oligo-pSc119.2 sites of 6RL belonged to linkage group 7 (**Figure 5**). Comprehensive FISH studies on cytogenetic stocks of 6RL deletions and genomic regions of the Lo7 genome have confirmed that the fine physical map of *Yr83* within FL 0.87-1.00 (806-881 Mb) was syntenic to linkage group 7. Therefore, the putative candidate genes for *Yr83*, and also for *Yr6R^Ar^L, YtSLU*, and *PmYT2*, might be syntenic with the functional conserved genes from the linkage group 7 of the Triticeae genomes.

Rye chromosome 6R contained the highest number of predicted NBS-LRR genes than any other rye chromosomes (Rabanus-Wallace et al., 2021). The 6RL distal region with homology to linkage group 7 region has accumulated NLR genes (Qian et al., 2021). Several genes for rust and powdery mildew resistance genes were characterized in the NLR rich gene clusters in the telomeric regions of wheat group 7 chromosomes, such as *Pm1* (Hewitt et al., 2021). Moreover, the *Pm60* gene (TraesCS7A02G553800) from wild emmer wheat (Zou et al., 2018; Wu et al., 2021) was also homologous to SECCE6Rv1G0450040, a NBS gene located at 865,626,675 to 865,630,526 bp of 6RL terminal region. Further investigation will be conducted on the expression of rye specific NLR genes in Triticale, wheat-rye addition and introgression lines under stripe rust challenge (Khalil et al., 2015; Zhao et al., 2022). The development of EMS mutants for the Sub6R(6D) and T6DS.6RL lines, and the subsequent target-sequence enrichment and sequencing (TEnSeq) pipeline for *Yr83* are undergoing (Zhang et al., 2020).

Substitution line Sub6R(6D) carrying *Yr83* conferred stripe rust resistance in China and Australia, and also displayed high levels of resistance to cereal cyst nematode and powdery mildew at adult plant stage (Dundas et al., 2001; Li et al., 2020a). We have shown that the 6R from Sub6R(6D) in a Sichuan wheat background has improved the agronomic traits, including the increase of spike length and reduced plant height (**Figure 1**). The translocation lines T6DS.6RL and T6AS.6RL as well as T7A-6R and T7D-6R (**Figure S2**) will be used as the starting lines for small translocation segment induction. The two Robertsonian translocations T6DS.6RL and T6AS.6RL might be able to be used directly in wheat breeding if no obvious linkage drag could be observed to introgress multiple disease resistances from rye 6R for wheat improvement.

## Data availability statement

The original contributions presented in the study are included in the article/**Supplementary Material**. Further inquiries can be directed to the corresponding authors.

## Author contributions

ZY, PZ, and ID conceived the project and designed the experiments. GL, QM, YZ, and JL performed the experiments, GL, YZ, and EY analyzed the data, ZY and GL wrote the paper. All authors read and approved the manuscript.

## Funding

The research was funded by the International Cooperation Project (2022YFH0012) of the Science and Technology Department of Sichuan, China, National Natural Science Foundation of China (31971886), and Guide Foundation for Research Institutions Performance Incentive in Chongqing (No. cstc2021jxjl80021).

## Acknowledgments

We are thankful to Jie Zhang (Biotechnology and Nuclear Technology Research Institute, Sichuan Academy of Agricultural Sciences) for helping us irradiate the seeds used in this study.

## Conflict of interest

The authors declare that the research was conducted in the absence of any commercial or financial relationships that could be construed as a potential conflict of interest.

## Publisher’s note

All claims expressed in this article are solely those of the authors and do not necessarily represent those of their affiliated organizations, or those of the publisher, the editors and the reviewers. Any product that may be evaluated in this article, or claim that may be made by its manufacturer, is not guaranteed or endorsed by the publisher.

## Supplementary material

Supplementary Figure 1 Sequential ND-FISH of T-701 **(A, D)** Sub6R(6D) **(B, E)** and T6DS.6RL line R23 **(C, F)** with Oligo-pSc119.2 + Oligo-pTa535 **(A, B, C)** and Oligo-248 **(D, E, F)** respectively. The arrows point to the FISH signal of Oligo-248 in the terminal region of 6RL.

Supplementary Figure 2 Different types of wheat-6R chromosome translocations were identified in the M3 progenies using ND-FISH. Oligo-Ku and Oligo-D **(A)** and Oligo-pSc119.2 + Oligo-pTa535 **(B)** were used to show chromosome T5DS.5DL-6RL **(A, B)**. Different types of translocations were showed in **(C)**, with breakpoints indicated by arrowheads **(C)**.

Supplementary Figure 3 The stripe rust responses of deletion lines R266, R367, and R76, translocation line R23, and substitution line Sub6R(6D). All three deletion lines were susceptible, whereas the translocation and substitution lines were highly resistant.

Supplementary Figure 4 FISH patterns of chromosome 6R by ND-FISH with Oligo-pSc119.2 (green) + Oligo-pSc200 (red) as probes. Three different karyotypes were observed for cultivated rye, which were distinctively different from *S. africanum*.
