# Supplementary figures and images for: Molecular and cytogenetic dissection of stripe rust resistance gene Yr83 from rye 6R and generation of resistant germplasm in wheat breeding
Source: Front Plant Sci. 2022 Oct 10;13:1035784. doi: 10.3389/fpls.2022.1035784 (PMC9589168; doi:10.3389/fpls.2022.1035784)

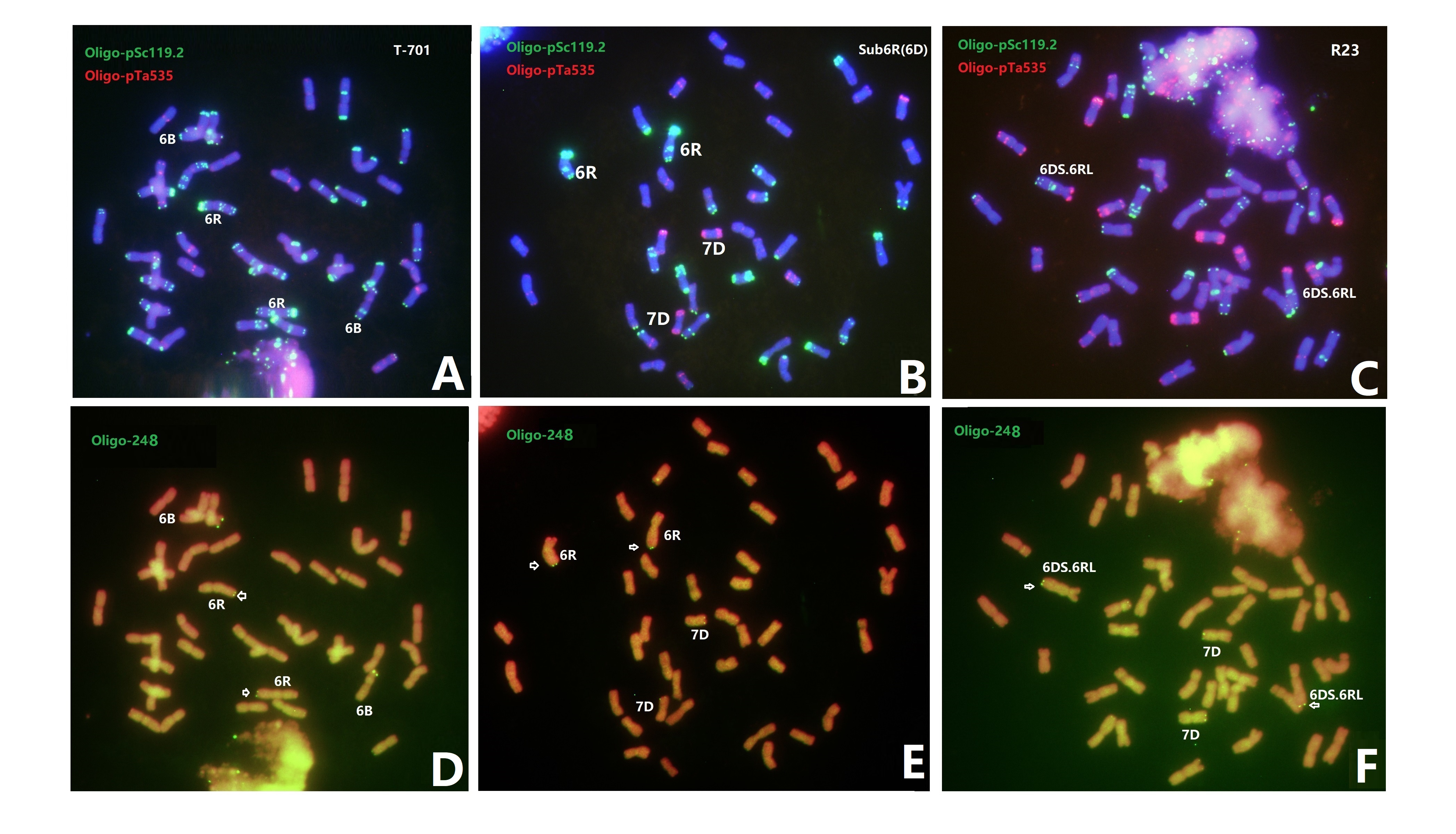

Supplement: Supplementary Figure 1 — Sequential ND-FISH of T-701 (A, D) Sub6R(6D) (B, E) and T6DS.6RL line R23 (C, F) with Oligo-pSc119.2 + Oligo-pTa535 (A, B, C) and Oligo-248 (D, E, F) respectively. The arrows point to the FISH signal of Oligo-248 in the terminal region of 6RL. [file Image_1.jpg]

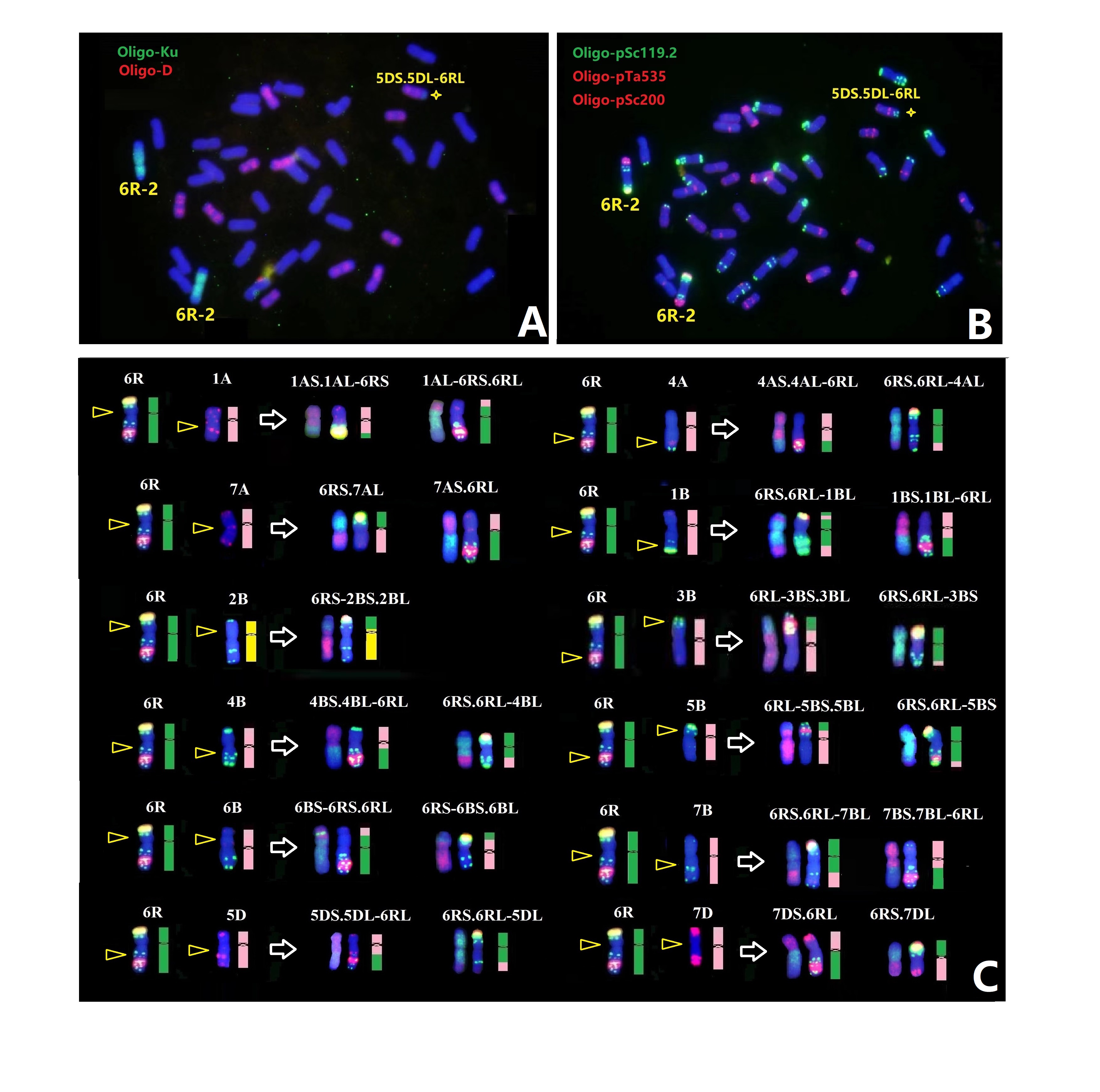

Supplement: Supplementary Figure 2 — Different types of wheat-6R chromosome translocations were identified in the M3 progenies using ND-FISH. Oligo-Ku and Oligo-D (A) and Oligo-pSc119.2 + Oligo-pTa535 (B) were used to show chromosome T5DS.5DL-6RL (A, B). Different types of translocations were showed in (C), with breakpoints indicated by arrowheads (C). [file Image_2.jpg]

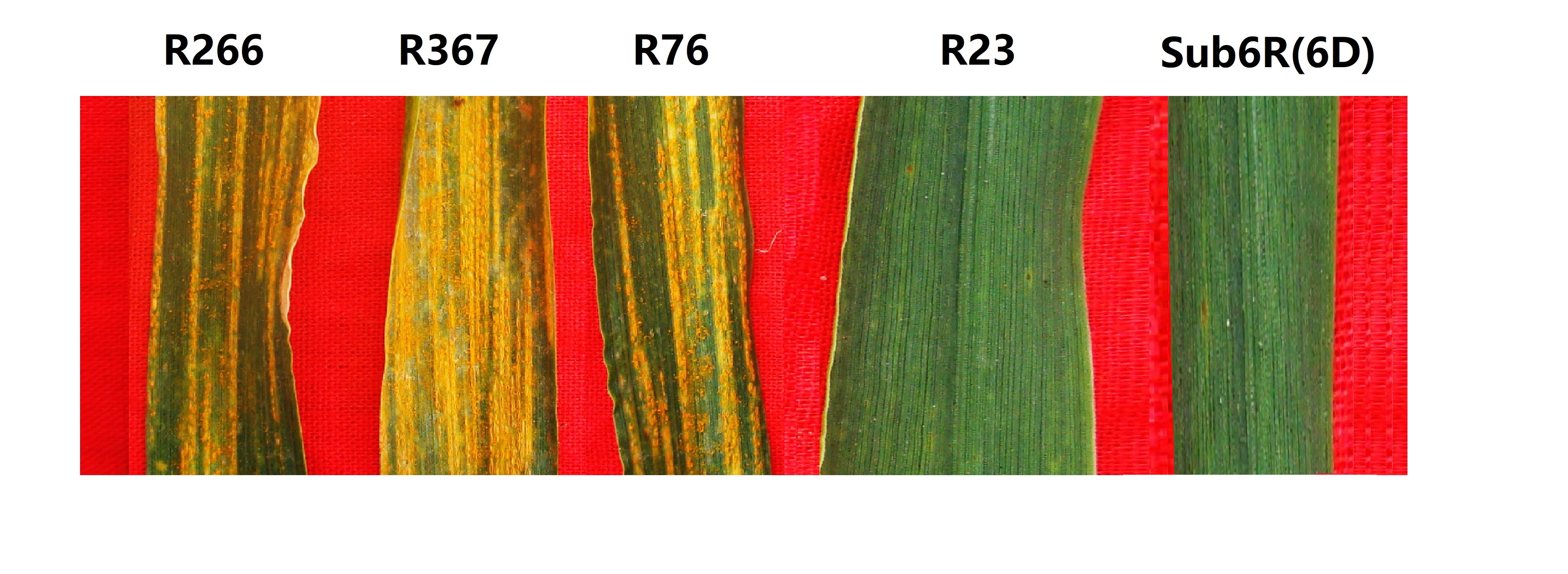

Supplement: Supplementary Figure 3 — The stripe rust responses of deletion lines R266, R367, and R76, translocation line R23, and substitution line Sub6R(6D). All three deletion lines were susceptible, whereas the translocation and substitution lines were highly resistant. [file Image_3.jpg]

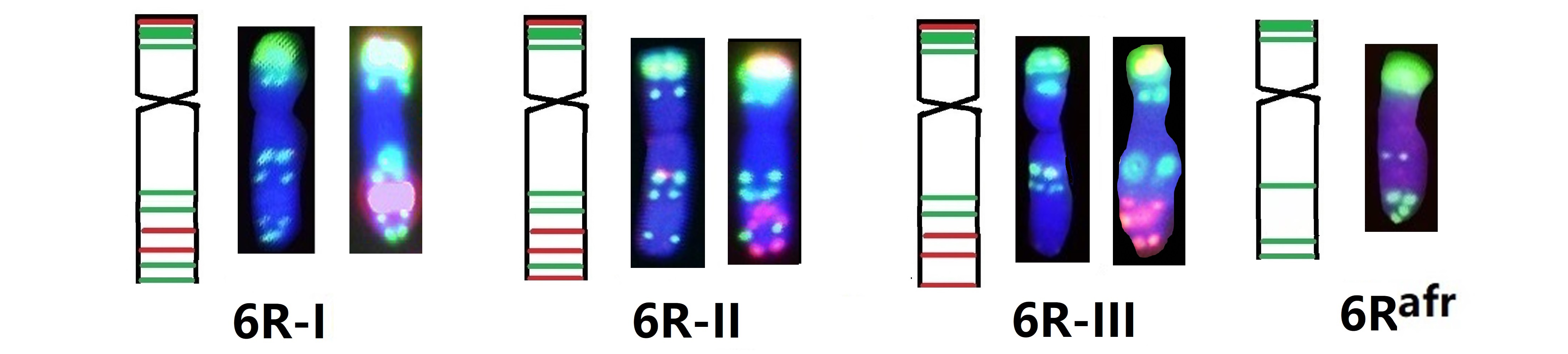

Supplement: Supplementary Figure 4 — FISH patterns of chromosome 6R by ND-FISH with Oligo-pSc119.2 (green) + Oligo-pSc200 (red) as probes. Three different karyotypes were observed for cultivated rye, which were distinctively different from S. africanum. [file Image_4.jpg]
